# Chorioamnionitis and Neonatal Sepsis from Community-associated MRSA

**DOI:** 10.3201/eid1512.090853

**Published:** 2009-12

**Authors:** Jason D. Pimentel, Frederick A. Meier, Linoj P. Samuel

**Affiliations:** Henry Ford Hospital, Detroit, Michigan, USA

**Keywords:** Chorioamnionitis, community-associated methicillin-resistant Staphylococcus aureus, staphylococci, MRSA, antimicrobial resistance, bacteria, letter

**To the Editor:** Chorioamnionitis is a common cause of maternal and neonatal illness and death (*1*), but chorioamnionitis attributed to *Staphylococcus aureus*, including methicillin-resistant *S. aureus* (MRSA), is reported infrequently (*2*–*5*). In the context of the rising incidence of community-associated MRSA (CA-MRSA) infections (*6*), we report an apparent case of CA-MRSA chorioamnionitis.

The patient, a 31-year-old woman with polycystic ovary syndrome and hypothyroidism, had 1 prior pregnancy but no viable offspring. After a clomiphene-assisted conception, routine ultrasound at 21 weeks’ gestation identified a shortened cervix (5 mm). The patient declined amniocentesis for cerclage and was treated with pelvic rest and vaginal progesterone. Five days later, she arrived at the emergency department with foul-smelling vaginal discharge. At this time, the patient was afebrile and hemodynamically stable, had no abdominal pain, and had a leukocyte count of 9.5 × 10^3^ cells/mm^3^.

Premature rupture of membranes was diagnosed, and the patient was admitted and administered intravenous ampicillin and azithromycin. Nine days into treatment, at 23 weeks’ gestation, 210 hours after membrane rupture, a 415-g live-born girl was delivered spontaneously in footling breech with Apgar scores of 1 (1 min) and 5 (5 min). During admission, the mother was never febrile and did not complain of abdominal tenderness or chills. The highest leukocyte count was 12.4 × 10^3^ cells/mm^3^. The mother was discharged the day after delivery without further complications. At 6-week follow-up, she remained well, with no signs of infection.

Pathologic examination of the placenta demonstrated focal acute funisitis, acute chorioamnionitis with fetal surface acute arteritis and acute deciduitis. Cultures from the maternal and fetal sides of the placenta grew predominantly MRSA and rare colonies of methicillin-susceptible *S. aureus*. The MRSA antimicrobial drug profile, including trimethoprim/sulfamethoxazole and clindamycin susceptibility, was characteristic of CA-MRSA (*6*).

The neonate, who died on day 16, was culture-positive for CA-MRSA from blood, 2 umbilical swabs, and a tracheal aspirate. The antibiogram of these isolates was identical to the placental cultures, including absence of inducible clindamycin resistance. Postmortem examination showed hemorrhagic necrotizing pneumonia and gram-negative bacilli. Culture of lung tissue grew *Escherichia coli*. Isolates from the placenta and neonate were identified phenotypically, without molecular testing.

Maternal complications of chorioamnionitis include endometritis, bacteremia, hemorrhage, and cesarean delivery (*1*). Clinically, chorioamnionitis can be diagnosed by maternal fever (>38°C) and 2 of the following: maternal leukocytosis (>15 × 10^3^cells/mm^3^), maternal tachycardia (>100 bpm), fetal tachycardia (>160 bpm), uterine tenderness, and foul-smelling amniotic fluid (*1*). This patient had none of these signs, except foul-smelling amniotic fluid, and fetal tachycardia was absent. In this case, chorioamnionitis was diagnosed by histology.

Amniotic fluid cultures from pregnancies complicated by chorioamnionitis have shown multiple organisms from the vaginal flora, such as *Streptococcus agalactiae*, *Gardnerella vaginalis*, *Mycoplasma hominis*, *Ureaplasma urealyticum*, anaerobes, and *E. coli* (*1*). Chorioamnionitis associated with *S. aureus* is uncommon (*2*,*3*), and MRSA chorioamnionitis is rare (*4*,*5*). The first 2 reports of MRSA chorioamnionitis appeared in 1998 (*4*) and 2002 (*5*). In both instances, the patients worked in the healthcare industry, and the authors considered the MRSA to have been nosocomial strains. The patient in our report was a restaurant manager, had no prior recorded hospital admissions, and was not previously known to be colonized by MRSA.

CA-MRSA strains are epidemiologically and clonally unrelated to hospital-associated MRSA (HA-MRSA) strains and can be differentiated by the presence of staphylococcal cassette chromosome *mec* type IV and the absence of multidrug resistance seen with HA-MRSA (*6*). Recently, the incidence of CA-MRSA infections increased in community settings, including outbreaks in settings in which CA-MRSA is endemic, with manifestations ranging from skin and soft tissue infections to necrotizing pneumonia (*6*). Genital colonization with MRSA recently has been reported with a frequency of 0.5%–3.5% in pregnant women (*7*,*8*). In 1 study, most (93%) of these isolates were CA-MRSA (*7*).

Eckhardt et al. described a patient with chorioamnionitis in whom CA-MRSA bacteremia developed (*9*). However, this descriptor was used to specify multidrug-resistant MRSA not acquired in a hospital. Moreover, neither placental nor amniotic fluid cultures were described. Laibl et al. reported 2 patients with CA-MRSA infections in whom chorioamnionitis developed (*10*). Again, placental and amniotic fluid culture results were not reported, nor was chorioamnionitis listed as an infection caused by CA-MRSA in their cohort. However, these latter 2 patients might represent additional cases of CA-MRSA chorioamnionitis.

Although the incidence of CA-MRSA infections continues to increase, CA-MRSA chorioamnionitis appears to remain rare. Nevertheless, the prevalence of MRSA genital colonization among pregnant women creates an opportunity for this agent to cause ascending gestational infection. This finding is meaningful because recommended empirical antimicrobial drug treatments may not cover CA-MRSA, increasing the likelihood of infectious complications (*1*). However, culture results when available can provide therapeutic guidance. We hope this report raises awareness of the possibility of CA-MRSA chorioamnionitis and encourages reports from other authors so this entity can be better established, characterized, and monitored.
